# The Role of Power Fluctuations in the Preference of Diagonal vs. Double Poling Sub-Technique at Different Incline-Speed Combinations in Elite Cross-Country Skiers

**DOI:** 10.3389/fphys.2017.00094

**Published:** 2017-02-21

**Authors:** Christine Dahl, Øyvind Sandbakk, Jørgen Danielsen, Gertjan Ettema

**Affiliations:** Centre for Elite Sports Research, Department of Neuromedicine and Movement Science, Faculty of Medicine, Norwegian University of Science and TechnologyTrondheim, Norway

**Keywords:** cross-country skiing, gross efficiency, kinematics, dynamics, technique

## Abstract

In classical cross-country skiing, diagonal stride (DIA) is the major uphill sub-technique, while double poling (DP) is used on relatively flat terrain. Although, the dependence of incline and speed on the preference of either sub-technique seems clearly established, the mechanisms behind these preferences are not clear. Therefore, the purpose of this study was to compare kinetics and energy consumption in DP and DIA at the same submaximal workload in cross-country skiing under two different incline-speed combinations. We compared kinetics and physiological responses in DP and DIA at the same submaximal workload (≈200 W) under two different incline-speed conditions, (5%—12.5 km h^−1^ vs. 12%—6.5 km h^−1^) where DP and DIA were expected to be preferred, respectively. Fifteen elite male cross-country skiers performed four separate 6.5-min roller skiing sessions on a treadmill at these two conditions using DP and DIA during which physiological variables, rate of perceived exertion (*RPE*) and kinetics, including power fluctuations, were recorded. At 12% incline, DIA resulted in lower physiological response (e.g., heart rate) and *RPE*, and higher gross efficiency than DP, whereas at 5% incline these variables favored DP (*P* < 0.05). The skiers' preference for sub-technique (13 preferred DIA at 12% incline; all 15 preferred DP at 5% incline) was in accordance with these results. Fluctuation in instantaneous power was lowest in the preferred sub-technique at each condition (*P* < 0.05). Preference for DP at 5% incline (high speed) is most likely because the speed is too high for effective ski thrust in DIA, which is reflected in high power fluctuations. The mechanism for preference of DIA at 12% incline is not indicated directly by the current data set showing only small differences in power fluctuations between DIA and DP. Apart from the low speed allowing ski thrust, we suggest that restricted ability to utilize the body's mechanical energy as well as the use of arms in DP play an important role.

## Introduction

Cross-country skiing is performed in varying terrain where skiers frequently make transitions between different sub-techniques. In the classical style, diagonal stride (DIA) is the major uphill sub-technique, and double poling (DP) is used for skiing on flat, as well as slight uphill or downhill terrain. During DIA, arms and legs follow a diagonal pattern, while generating propulsive forces relatively continuously (Kehler et al., 2014) with long poling times emphasizing force production in the later part of the poling cycle (Lindinger et al., 2009). One of the advantages of DIA therefore lies in the high “duty factor,” i.e., an almost continuous propulsion action by alternating ski and pole propulsion by the left—and right body sides. Likely, this leads to low fluctuations in power and speed during each cycle. In DP, all propulsion comes through the poles since the skis glide continuously forward. Compared with DIA, the production of propulsive forces in DP is characterized by a relatively short poling time with a rapid force increase through propulsion actions from both poles simultaneously (Holmberg et al., 2005; Pellegrini et al., 2013; Danielsen et al., 2015; Stöggl and Holmberg, 2016). Thus, DP has much lower duty factor, characterized by discontinuous propulsion from symmetrical left and right pole strokes. In DIA, speed is restricted by the requirement of a stationary moment in the ski movement that allows the generation of ski propulsion forces (Lindinger et al., 2009). Of course, the same principle applies to the poles that are fixed on the ground during propulsion, but the link from hips to the tips of the poles has a larger range of motion, allowing for higher skiing speed at which body movement speed becomes restricted. This leads to increased demands on upper body power generation at higher speed. Such restriction on ski thrust does not occur in DP. The significance of the mechanical advantages and disadvantages of DP and DIA, and thereby the preference for one sub-technique, likely depend on both speed and incline. At slight inclines with high speeds leading to high extension speed of the lower extremity and restricting propulsion in DIA, one would expect preference for DP. At low speeds and steep inclines, the high duty factor as well as the ability to propel force through both skis and poles may be the reason for the DIA preference. It should be noted that during races, other factors such as fatigue and/or pacing tactics also will affect the choice of sub-technique at a given incline and speed.

Since skiing speed and terrain incline are inversely linked to each other at constant work rate, the effect of incline, speed, and work rate are tightly entangled. In previous studies of the selection and effectiveness of sub-technique, either speed or incline is changed, keeping the other constant. This leads to considerable changes in work rate. For example, Pellegrini et al. (2013) investigated this by comparing different inclines at one speed and different speeds at one incline. At 10 km/h, preference for DP shifted to DIA at an incline of 2–3 degrees. At a 2° incline, most athletes preferred DP, and DIA only by some skiers at the lowest speeds (up to 8 km h^−1^). While their findings may support the notion about effect of speed and incline, it is difficult to distinguish from the effect of changing work rate. The same applies to the comparison of physiological load of two ski-skating sub-techniques under different slopes and speeds (Kvamme et al., 2005).

In these studies, work rate may have had a significant impact on the gross efficiency outcome simply because of the zero work rate offset, i.e., humans have considerable energy consumption at rest or zero power production (Ettema and Lorås, 2009). Thus, one must be cautious when comparing different sub-techniques in cases that both different work rates and metabolic rates have occurred. To date, studies on the effect of incline and speed that are independent of workload in the comparison of DP and DIA are non-existent in the literature. Although, the dependence on incline and speed for the preference of either sub-technique seems clearly established (Bilodeau et al., 1996; Pellegrini et al., 2013; Andersson et al., 2016), the reasoning and mechanisms behind these preferences are not clear, especially when considering the relation between sub-technique and energy consumption.

The aim of this study was to compare kinetics and energy consumption and energy consumption in DP and DIA at the same submaximal workload in cross-country skiing under two different conditions, i.e., slight incline—high speed and steep incline—low speed, where DP and DIA were expected to be preferred, respectively. This was executed while roller skiing on a treadmill by examining both energy expenditure and aspects of sub-technique relating to continuity of propulsion. It was expected that preference of either sub-technique related closely to the energy expenditure and technical characteristics (e.g., duty factor, fluctuations in power and speed, distribution of power generated through the poles and skis).

## Methods

### Participants

Fifteen elite male cross-country skiers competing at national and international level (age 24.0 ± 2.7 years, body height 182.6 ± 4.6 cm, body mass 76.4 ± 6.4 kg) volunteered to participate in this study. All skiers were familiar with treadmill roller skiing from previous training and testing. The study was registered, and approved by Norwegian Social Science Data Services. Prior to obtaining written informed consent, the protocol and procedures were explained both in writing and verbally to each subject individually, and were also informed explicitly that they could withdraw at any time without giving any reason. The study was conducted in accordance with the Declaration of Helsinki.

### Experimental design

The participants were asked to adhere to their standard diet as under training and competition, and not make alterations during the period of testing. All participants completed a 15-min low intensity warm-up, which was self-paced and standardized according to the warm-up approach used by these athletes in training and competition. Warm-up was done roller skiing on the same inclines as used in the main experiments. Apart from athlete preparation, this ensured that the roller ski wheels and bearings reached a proper temperature (Ainegren et al., 2008).

To investigate DP and DIA in different experimental conditions two inclines (moderate 5% and steep 12%) were selected and two speeds (12.5 and 6.5 kmh^−1^) were found to obtain an approximately 200 W workload. This workload was chosen to obtain an intensity just below lactic threshold for these athletes, ensuring aerobic steady state conditions. The speeds were estimated based on average body mass and taking into account mass dependent roller friction power. In a pilot study, it was established that the athletes could perform both sub-techniques comfortably. All participants performed four test sequences (two incline-speed combinations × two sub-techniques), in a randomized order with a minimum of a 5-min recovery between sequences. Each test sequence consisted of a 6.5-min bout of steady-state submaximal roller skiing. Respiratory variables and heart rate (*HR*) were averaged over the last 2 min to ensure steady state conditions. Lactate blood samples were taken prior and immediately after this period (during a short break from exercise, while standing on the treadmill). Afterwards, the athletes continued for about 90 s at the same incline and speed, during which pole forces and kinematics were recorded in the last 75 s. Respiratory and kinetics recordings were done separately to avoid interference of these recordings. The athletes were only instructed on the sub-technique to use (DP or DIA), and otherwise were left free in the technical execution.

The actual work rate was calculated as power against gravity and roller friction estimated, taking unloading of skis by pole force into account, according to Sandbakk et al. (2012)—see below for details. Aerobic metabolic rate was determined from gas exchange, and blood lactate values were used to ensure that submaximal aerobic conditions were maintained (lactate below 4 mmol). Gross efficiency (*GE*) was calculated as the work rate divided by the aerobic metabolic rate. Directly after each sequence, rating of perceived exertion (*RPE*) was recorded as well as the sub-technique (DP or DIA) the athlete would have preferred.

### Instruments and materials

Roller skiing was performed on a 5 × 3-m motor driven treadmill (Forcelink Technology, Zwolle, The Netherlands). Incline and speed were calibrated using the treadmill software. To minimize variations in rolling resistance, all of the skiers used the same pair of roller skis that ensure full grip in the DIA sub-technique with standard wheels with resistance category 2 (IDT Sports, Lena, Norway). The surface of the treadmill belt was covered with non-slip rubber and the participants used poles with special carbide tips, available at incremental lengths of 5 cm (Madshus UHM 100, Biri, Norway). The athletes could choose their own pole length but not alter this between tests. The athletes were secured with a safety harness connected to an emergency brake. The rolling friction coefficient (μ) of the roller skis was determined three times for both speeds used in this study by a towing test at the start and end of the experiments as described previously by Sandbakk et al. (2010). The overall mean value of μ was 0.018 ± 0.001, and included in the calculation of work rate.

### Physiological measurements

Respiratory variables and oxygen consumption (*VO*_2_) were measured continuously by an open circuit indirect calorimetry using an Oxycon Pro apparatus (Jaeger GMbH, Hoechberg, Germany). At the beginning of each test day, the system was calibrated against a known mixture of gases (16.00 ± 0.04% O_2_ and 5.00 ± 0.1% CO_2_, Riessner-Gase GmbH & Co, Lichtenfels, Germany), and the expiratory flow meter was calibrated with a 3 L volume syringe (Hans Rudolph Inc, Kansas City, MO). *HR* was recorded using a Polar heart rate monitor (V800 Polar Finland). Blood lactate values was obtained from a 20 μl blood sample collected from the middle and ring finger, and analyzed using Biosen C_line Sport lactate analyzer (EKF-diagnostic GmbH, Barleben, Germany). The aerobic metabolic rate was calculated as the product of *VO*_2_ and the oxygen energetic equivalent using the associated respiratory exchange ratio (*RER*) and standard conversion tables (Péronnet and Massicotte, 1991). Gross efficiency was calculated as the external work rate performed by the entire body divided by the aerobic metabolic rate. *RPE* was assessed using the Borg Scale (Borg, 1970) for whole (*RPE*_*wb*_), lower (*RPE*_*lb*_), and upper body (*RPE*_*ub*_).

### Dynamics

Pole forces were measured by two CDF Miniature Button Load Cells (diameter, 15 mm; height, 8 mm; capacity, 2,000 N; non-linearity, <0.5%; hysteresis, <0.5%; weight, 10 g; Applied Measurements LTD, Aldermaston, Berkshire, UK) mounted in both poles. The load cells were placed on top of an aluminum (50 g) tube, which was mounted directly at the top of and inside the pole tube. A small (8 mm diameter) ball was located in between the load cell and the aluminum tube which minimized the cross-talk between forces directed along the pole and forces associated with squeezing, bending, or rotation of the hand grip. The pole force measurement device was calibrated against a force platform (Kistler 9286AA, Kistler Instruments, Winterthur, Switzerland) and validity for poling was checked by applying forces in a poling like action. The maximal error during peak force exertion was 5 N. Pole forces were sampled at 1,500 Hz and recorded via a telemetric system (TeleMyo DTS, Noraxon, Scottsdale, AZ, USA).

### Kinematic measurements

Nine Oqus infrared cameras (Qualisys AB, Gothenburg, Sweden) captured three-dimensional position characteristics of passive reflective markers at a sampling frequency of 250 Hz. Each recording session lasted ~75 s, which ensured at least 30 poling cycles for each test sequence. The coordinate system was calibrated according to the manufacturer's recommendation. The same researcher positioned passive reflective markers on anatomical landmarks bilaterally by using double sided tape (3 M, USA). These landmarks were on the boot at the distal end of the fifth metacarpal, the lateral malleolus (ankle), lateral epicondyle (knee), greater trochanter (hip), lateral end of the acromion process (shoulder), lateral epicondyle of humerus (elbow), styloid process of ulna (wrist), and C7 (neck). A total of 8 markers were placed on the poles and roller-skis. One marker was placed on the lateral side of each pole, 5 cm below the handle, and one marker placed on the lateral side of the pole tip. These markers allowed for calculating pole direction and thus direction of pole forces. Two markers were fixed on the left side of the treadmill in alignment to movement direction with a 1-m distance so that belt incline was recorded throughout the protocol. Two markers were attached on each ski, one marker 1 cm behind the front wheel, and one marker 1 cm in front of the back wheel of each roller ski. Acquisition software (Qualisys Track Manager, Qualisys AB, Gothenburg, Sweden) was used to synchronize dynamics and kinematics, and further data analysis was done using in a purpose-written script in Matlab (R2014a, Mathworks Inc., Natick, MA, USA). All position data were low-pass filtered (Chebyshev II filter, 8th order, 20 Hz cut-off) before further processing. Body center of mass (*CoM*) was determined from these position data and body segments masses (including skis and poles) according to de Leva (1996). *CoM*'s velocity was obtained by numerical differentiation of horizontal and vertical *CoM* position data. Orientation of the poles was used to determine the pole force vector for calculations of pole power.

### Data analysis

**Figure 1:**
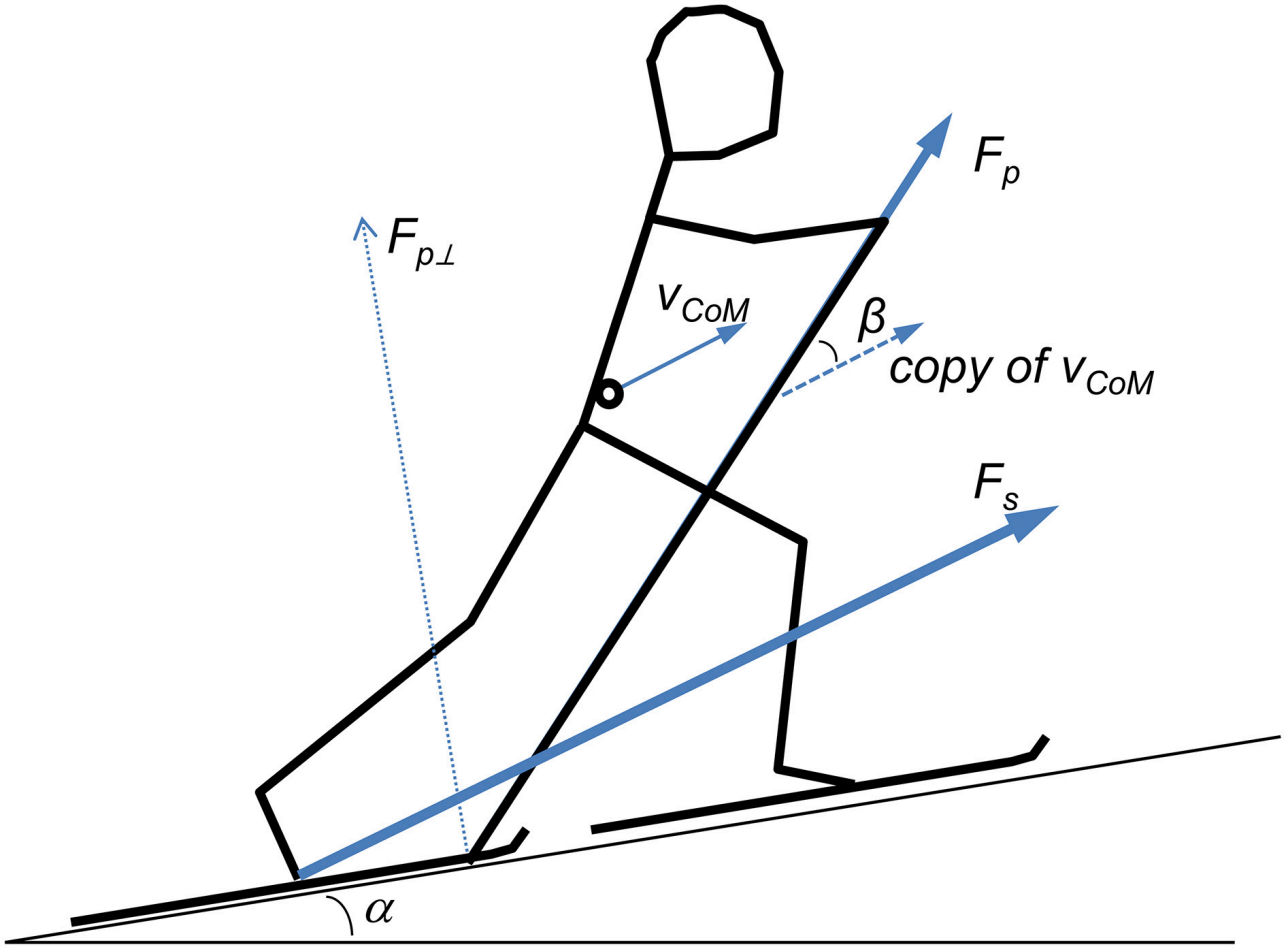
**Stick diagram showing key vectors for pole force and—velocity while roller skiing**. *F*_*s*_, ski force; *F*_*p*_, pole force; *F*_*p*⊥_, pole force perpendicular on ground surface; *V*_*CoM*_, velocity of center of mass; α, angle of incline; β, angle between *F*_*p*_ and *V*_*CoM*_.

Movement variables that were used in statistical analysis were based on kinematic and kinetic data described above. Propulsion periods for the poles were defined as the period when the poles were in contact with the belt. Belt contact was identified by comparing the pole velocity vector of the distal pole marker with belt velocity vector, i.e., when movement direction and velocity magnitude of pole marker and belt are (close to) identical. For DIA, ski propulsion periods were identified by a similar procedure, comparing ski movement relative to the belt. For DP, ski propulsion times were set at zero, by definition. Total propulsion time was defined as the time that any of the poles or skis was in state of propulsion as defined above. Duty factor (*DF*) was defined as the ratio between total propulsion time and cycle time. For cycle identification, the left pole plant was used and identified by the onset pole-belt contact. Cycle rate (*CR*) was calculated as the reciprocal of the average time between left pole plants. Cycle length was not taken into consideration because it is mathematically fully dependent on *CR* and protocol determined speed. Mean external work rate (*P*_*mean*_) was calculated in accordance to Sandbakk et al. (2010), as the sum of power against gravity and friction:

(1) $$\begin{array}{r} P_{mean}=mg\;v\;sin\alpha +\left(mg\;cos\alpha -F_{p⊥}\right)\mu v, \end{array}$$

where *m* is the body mass of the skier, *g* the gravitational constant, α inclination of the treadmill, *v* the speed of the treadmill, *F*_*p*⊥_ the mean pole force perpendicular to the belt (see Figure 1), and μ the frictional coefficient.

Poling power (*P*_*p*_) was calculated as the dot product of pole force vector and CoM's velocity vector:

(2) $$\begin{array}{r} P_{p}=F_{p}v_{CoM}\cos\beta \end{array}$$

*F*_*p*_ and *v*_*CoM*_ are magnitudes of the pole force vector and *CoM*'s velocity vector (relative to the belt's speed), respectively, and β the angle between these vectors (Figure 1). While power fluctuations can be established for DP because all propulsion forces are recorded (pole propulsion only), this is not the case for DIA. However, given that instantaneous power is used for three purposes, power against gravitational losses, against roller friction, and driving changes of kinetic energy (van Ingen Schenau and Cavanagh, 1990; de Koning et al., 2005), fluctuations in instantaneous power could be approximated using CoM's speed fluctuations. Instantaneous power is:

(3) $$\begin{array}{r} P_{i}=P_{g}+P_{f}+\frac{dE_{kin}}{dt}=P_{g}+P_{f}+mv_{i}a_{i} \end{array}$$

This can be approximated using Equation (1) as:

(4) $$\begin{array}{r} P_{i}=\;v_{i}\;\left(mg\;sin\alpha +\left(mg\;cos\alpha -F_{p⊥}\right)\mu +ma_{i}\right) \end{array}$$

With *v*_*i*_ and *a*_*i*_, velocity and acceleration of CoM in goal direction, respectively, and *P*_*i*_ instantaneous power associated to this movement in goal direction. The maximal changes in *P*_*i*_ during one cycle (Δ*P*) were used to quantify fluctuations.

### Statistical analysis

All data were checked for normality using the Shapiro–Wilk test and inspection of q-q plots. Two-way (2 × 2) repeated measures ANOVA was used to analyze effects of condition, sub-technique, and the interactions. *Post-hoc* tests with Bonferroni corrections were performed to locate differences. The level of statistical significance was set at α = 0.05. All analyses were performed with Statistical Package for the Social Sciences (SPSS 22; IBM Corp., Armonk, NY).

## Results

**Figure 2:**
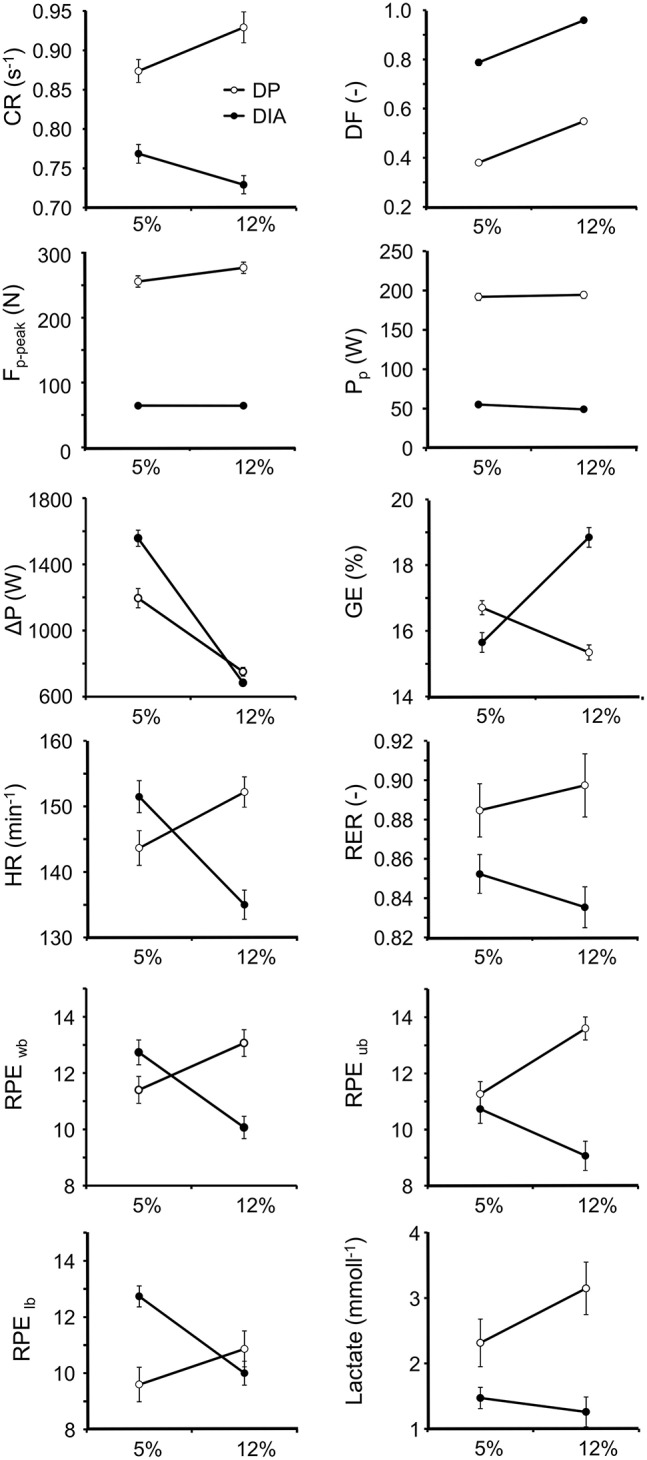
**Comparison of kinetics, physiological responses, and perceived load (mean and s.e.m., ***N*** = 15) in DIA and DP in two incline-speed conditions at 200 W workload while roller skiing**. The statistical outcome of the comparisons is given in Table 1. *CR*, cycle rate; *DF*, duty factor (fraction of cycle time); *F*_*p*−*peak*_, peak pole force; *P*_*p*_, pole power; Δ*P*, power fluctuation. *GE*, gross efficiency; *HR*, heart rate; *RER*, respiratory exchange ratio; *RPE*, rate of perceived exertion; *wb*, whole body; *ub*, upper body; *lb*, lower body.

All results are depicted in Figure 2, and the corresponding statistical outcome in Table 1. Sphericity was never violated and thus no corrections were needed for this. The findings, which are presented in detail below, are in strong agreement with the preference indicated by the athletes. All athletes preferred DP at 5%, 13 of 15 athletes preferred DIA at 12% (binomial test: *p* < 0.001). None of the athletes indicated to prefer DP with kick, the sub-technique between DIA and DP (Pellegrini et al., 2013). Two athletes preferred DP over DIA in any condition. These two athletes did not differ from the group with regard to any of the other variables and were therefore included in the statistical analysis. In DIA about 50 W (~25%) of total power is delivered through the poles at a lower *CR* than in DP, irrespective of the condition. The relatively small difference for pole power contribution between incline-speed conditions for DIA (28 vs. 25% of total power) was significant (*p* = 0.004). *DF* in DIA exceeds *DF* in DP by a value of 0.41 (*p* < 0.001; Table 1, Figure 2) and approaches 1 at 12% incline. In both sub-techniques, a similar effect of incline-speed condition was found for *DF*. On average, *GE* was higher in DIA, mostly because of the relatively high value at 12% incline. Condition affects *GE* strongly, and in an opposing direction for the two sub-techniques (Figure 2). GE, physiological response (*HR* and *RER*), and *RPE* are coherent and indicate aerobic conditions below lactate threshold (see Figure 2; Table 1). *HR* amounted to 75 ± 5% (s.d.) of maximal heart rate. Particularly, *HR* and *RPE*_*wb*_, and to lesser extent *RPE*_*lb*_ show the same (but reversed) pattern as *GE*. *RER* and *RPE*_*ub*_ values also agree with this, but in DP, these variables are larger than DIA irrespective of condition. Δ*P*_*i*_ showed a similar but reversed pattern as *GE*. At 5%, DP shows less fluctuation than DIA, which was the opposite at 12%.

**Table 1:** **Statistical outcome, i.e., ***p***-values (η^**2**^ for two way ANOVA; Cohen's d for local differences) of the 2 × 2 ANOVA for repeated measures [***N*** = 15, ***df***_**(1, 14)**_] with Bonferroni corrections for the local differences**.

**Variable**	**Two way ANOVA**			**Local differences**			
	**Condition**	**Sub-technique**	**Interaction**	**Sub-tech. at 5%**	**Sub-tech. at 12%**	**Cond.in DIA**	**Cond.in DP**
*P_*mean*_*	(0.79)^**^	(0.99)^**^	0.678 (0.01)	(0.19)^**^	(0.19)^**^	(0.16)^**^	(0.16)^**^
*CR*	0.459 (0.04)	(0.87)^**^	(0.62)^**^	(2.04)^**^	(3.21)^**^	(0.86)^**^	0.014 (0.83)
*DF*	(0.98)^**^	(0.99)^**^	0.85 (0.003)	(9.76)^**^	(17.45)^**^	(4.38)^**^	(6.08)^**^
*F_*p*−*peak*_*	0.016 (0.37)	(0.98)^**^	(0.35)^*^	(07.71)^**^	(8.45)^**^	0.97 (0.01)	0.013 (0.62)
*P_*p*_*	0.090 (0.21)	(0.98)^**^	(0.61)^*^	(9.91)^**^	(11.40)^**^	(0.73)^*^	(0.15)^**^
*ΔP* in cycle	(0.97)^**^	(0.55)^*^	(0.89)^**^	(1.73)^**^	(0.73)^*^	(6.02)^**^	(2.53)^**^
*GE*	(0.69)^**^	(0.68)^**^	(0.94)^**^	(0.98)^*^	(3.30)^**^	(2.76)^**^	(1.57)^**^
*HR*	(0.51)^*^	(0.55)^*^	(0.87)^**^	(0.79)^**^	(1.96)^**^	(1.82)^**^	(0.89)^**^
*RER*	0.485 (0.04)	(0.62)^**^	(0.48)^*^	(0.71)^**^	(1.18)^**^	(0.43)^*^	0.028 (0.22)
*RPE_*wb*_*	0.241 (0.10)	0.052 (0.24)	(0.70)^**^	0.027 (0.75)	(1.78)^**^	(1.64)^**^	(0.91)^*^
*RPE_*ub*_*	0.344 (0.06)	(0.88)^**^	(0.52)^*^	0.292 (0.29)	(2.49)^**^	0.034 (0.83)	(1.41)^**^
*RPE_*lb*_*	0.083 (0.20)	0.053 (0.24)	(0.51)^*^	(1.59)^*^	0.263 (0.41)	(1.77)^**^	0.078 (0.52)
*Lactate*	(0.73)^**^	(0.68)^**^	(0.50)^*^	(0.77)^*^	(1.50)^**^	0.182 (0.28)	(0.56)^**^

P < 0.001 and P < 0.0125 are indicated by ^**^ and ^*^, respectively. P_mean_, mean external power; CR, cycle rate; DF, duty factor; F_p−peak_, peak pole force; P_p_, pole power; ΔP, power fluctuation in one cycle (maximum − minimum); GE, gross efficiency; HR, heart rate; RER, respiratory exchange ratio; RPE, rating of perceived exertion; wb, ub, lb: whole-, upper-, and lower-body, respectively. Means and s.e.m. are presented in Figure 2.

**Figure 3:**
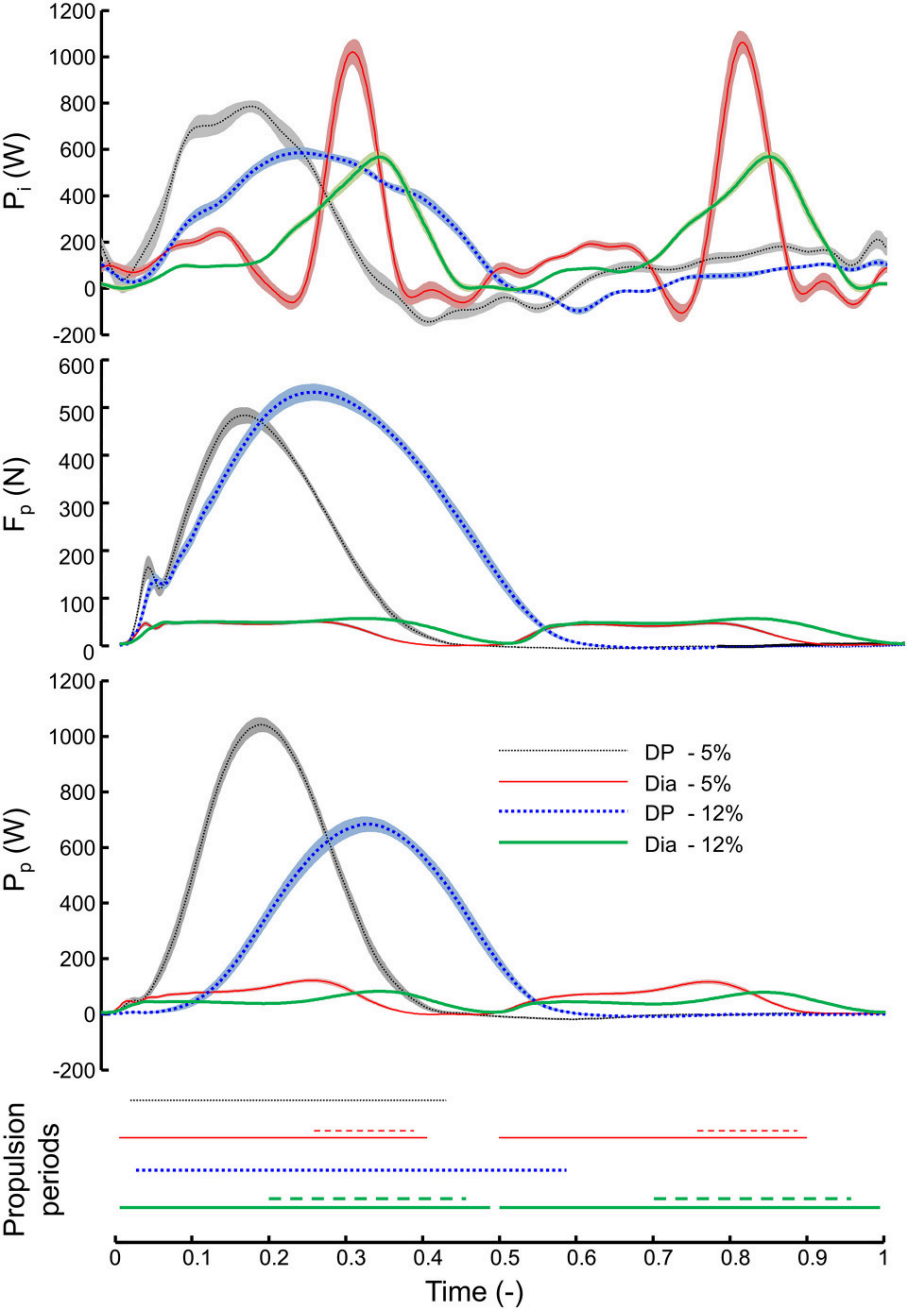
**Traces (mean and s.e.m., ***N*** = 15) of instantaneous external power, pole force, and pole power against normalized time (i.e., 1 is full cycle) while roller skiing**. Lower diagram indicates the periods of thrust for poles (solid or dotted lines) and skis (dashed lines). Pole force and power are the sum of left and right. *P*_*i*_, instantaneous total power; *F*_*p*_, pole force; *P*_*p*_, pole power.

Time traces for total and pole specific power, as well as propulsion periods are presented in Figure 3. It shows that *DF* is fully determined by pole propulsion; in DP this is obvious and in DIA the appearance of ski propulsion does not affect *DF* because it overlaps fully with pole propulsion. However, the in-phase (DP) vs. out-of-phase (DIA) propulsion leads to much higher *DF* values in DIA (Figures 2, 3). Accounting for this (Figure 3 bottom), relative duration of pole propulsion is similar in DP (0.368) and DIA (0.363) at 5%, but at 12% DP has relatively longer lasting pole propulsion (0.536 vs. 0.483). In absolute time, pole propulsion lasts longer in DIA than DP. Otherwise, absolute and relative propulsion show the same qualitative differences as depicted by the relative values (Figure 3 bottom). The traces for total power in DIA divert strongly from those in DP; the large peak power generations in DIA at a 5% incline coincide with the ski propulsion periods, and are the reason for the large power fluctuations for this condition (Figure 2).

External work rate deviated somewhat from the 200 W target because of difference between estimated and actual body mass and incorporation of unloading of the skis by pole forces in the final calculation. External power amounted on average to 195.5 ± 16.2 (s.d.) Watt. The maximal difference between the four conditions for one individual was 8.6 W. It should be noted that while the differences were significant (see Table 1), they were relatively small. The inter-individual differences are mainly due to difference in body mass.

## Discussion

The current study aimed to investigate the underlying mechanisms behind the preferences for either DP or DIA at two different combinations of incline and speed at similar external work rate. It was expected that lower physiological response (*HR, RER*), lower *RPE* values, and higher *GE* were associated with low power fluctuation and high *DF* in DIA at 12% incline and in DP at 5% incline, respectively. These expectations were mostly confirmed and in agreement with preference for DIA at 12% and DP at 5% incline. The 5% incline condition showed a lower *HR, RPE*_*wb, lb*_, and higher *GE* for DP than in DIA, while power fluctuations were lower in DP than in DIA. At 12%, DIA was preferred, possibly due to higher *GE* and lower physiological response and RPE as well as the lowest power fluctuations of all conditions tested. *RPE*_*ub*_, *RER*, and lactate are generally higher in DP (indicating a higher demand on the upper extremity), but show reversed differences between incline-speed conditions compared to DIA, which support the other findings in our study. In addition, for DIA, it was expected that incline-speed condition would affect the division of power between poles and ski in relationship with preference and physiological response. The difference in pole power contribution was relatively small (25% at 12% incline vs. 28% at 5% incline) but indeed significant, and indicated that at slight incline and high speed, the lower limb contributed less than at steep incline.

*DF* is not an explanatory factor for preference and physiological response by itself, because both sub-techniques show almost identical differences between conditions. However, *DF* coincided with speed fluctuations that form the basis of power fluctuations (Equation 4). Thus, the increase in *DF* in both sub-techniques from 5 to 12% incline is likely a contributing factor for the associated decrement of power fluctuations at 12% incline. It should be noted that, as opposed to *DF*, power fluctuation shows statistical interaction effects, i.e., it can be an explanatory factor for the same interactions in preferences and physiological response in DP and DIA at both incline-speed combinations.

The striking characteristics of DIA at 5% incline (high speed) are the large power fluctuations with short bursts of peak power occurring at the time of ski-thrust (Figure 3), which are much less apparent at 12% incline (low speed). The most likely explanation for this is that, at high skiing speed, the lower extremity has a relatively narrow time-slot at which it must move at high extension speed, basically matching the skiing speed. Pellegrini et al. (2013) suggest that thrust times ideally should be >0.22 s based on the time it takes for muscle force development. Thrust times for DIA in our study were 0.2 s (5% incline) and 0.36 s (12% incline) on average. Thus, our findings are in agreement with Pellegrini et al. (2013) and suggest that the 5% incline condition is performed at a speed just too fast for optimal ski thrust in DIA. Note that in Pellegrini et al. (2013), skiers may have preferred double poling with kick instead of DP (current study) under this condition. Of course, the same principle applies for upper extremity, but the link from the hips to the tips of the poles involves a greater number of joints and body segments (including poles). The maximal movement speeds in these joints will therefore likely be reached at higher skiing speeds than in the lower extremity. In this regard, it is of interest to test maximal movement speeds of the upper and lower body in unloaded imitation movements mimicking DIA and DP.

The *CR*—and *GE* differences between conditions in one sub-technique may suggest that *CR* has a negative effect on *GE* and is an explanation for differences in physiological response. However, the athletes were free in the choice of *CR*, which therefore cannot be regarded as a direct cause. For example, in DIA, *CR* is highest at 5%, but likely in an attempt to create the best conditions for ski propulsion, and yet, still leading to high power bursts and reduced *GE*.

At 12% incline, power fluctuations in DIA are less than in DP. In combination with sub-technique preference and physiological response, this supports our hypothesis that power fluctuations are a primary factor in sub-technique choice. However, the differences in power fluctuations between sub-techniques seem too small to explain the large *GE* differences; particularly that *GE* in DIA is very high. In other words, the physiological results and *GE* provide a good clarification for the preference of DIA at 12% incline, yet, power fluctuations do not seem to be the variable that indicates the mechanism behind this finding. Thus, other reasons may be considered. In DP, the use of lower extremity (i.e., increasing body's energy during recovery phase, which is then re-utilized in pole propulsion; Danielsen et al., 2015), may be impaired at steep inclines. Stöggl and Holmberg (2016) find that in DP, the center of mass is raised more in steep uphill than in flat terrain, which does not agree with this notion, but this study used a different combination of speeds and inclines than the current study, and most importantly, did not compare combinations with similar external work rate. Alternatively, the active use of the arms may be limited because of positioning of the body above the support area when moving on a slope (see Stöggl and Holmberg, 2016), which affects the movement range for poling. The increasing effect of incline on *CR* during DP, which is in line with findings by Millet et al. (1998) and Stöggl and Holmberg (2016) may be the way to uphold use of the arms to some extent. In any case, in DP at 12% incline, the athlete may have to rely more on a smaller muscle mass to generate power than at 5% incline. This notion is supported by the *RPE*_*ub*_ values.

Although, the efficiency in DIA at 12% incline is much higher than in the other three conditions, it does not necessarily mean that in DP one cannot reach the same high efficiency under other conditions than examined in this study. This may actually be the case at higher speeds than analyzed in the present study. For example, Andersson et al. (2016) found efficiency values for DP that were higher at higher speeds than in our study. It should be noted that they, as we did, also found higher peak efficiencies in DIA than in DP.

If power fluctuations are indeed a primary factor for sub-technique preference, then it is likely that power fluctuations can determine the preference of DP at 5% incline. Between incline-speed conditions, the sub-technique preference and low physiological response coincides with, and thus seems to be explained by, minimizing power fluctuations. It should be noted that at 12% incline the DIA-DP differences are small. Moreover, within one sub-technique, the effect of condition on the changes in mechanical characteristics and in corresponding physiological response is not consistent with this. For example, if power fluctuations would explain all, then physiological response would be lower at 12% incline in DP as it is in DIA. This is clearly not the case; thus, more than one factor must be considered to explain physiological response.

A similar rationale applies to the determinants of transition of sub-techniques when changing incline or speed. For example, Pellegrini et al. (2013) followed the rationale presented by Hreljac (1995), and argued that pole forces are a key determinant for transitioning to the preferred sub-technique which may be coupled with an increased energy cost in arm-only propulsion with DP. However, the arguments raised by them (Pellegrini et al., 2013) should be considered with caution since power in DP is generated about 50% by the lower body (Danielsen et al., 2015) despite that propulsion only occurs via the poles. Our results on poling forces and associated power fluctuations in DP also contradict this notion. Specifically, peak poling forces in DP at 12% incline are only marginally higher than at 5% and exceed pole forces in DIA at both inclines by far. Note that in Pellegrini et al. (2013) the comparison is made between different work rates (increasing incline at given speed), which logically will lead to higher propulsive forces. In a wider context, it is futile to search for a single determinant for transitions of locomotor mode. More likely, in endurance sports, one may consider various determinants that relate to minimization of energy expenditure.

The four combinations of incline-speed and sub-technique resulted in small differences in external workloads (Table 1). The unloading of skis by pole forces and its effect on roller friction could not be estimated beforehand, and thus, in this type of experiment it is almost impossible not to create small differences in load between conditions and sub-techniques. Also, possible changes in μ because of changes in normal force (Ainegren et al., 2008; by about 0.002 in the present study) may have affected the true work rate, but only marginally. Overall, such differences were minor and can hardly have attributed to the current findings with regard to mechanical characteristics and physiological response.

In summary, the current study confirms that DP and DIA are preferred sub-techniques at different combinations of incline and speed (12%-slow for DIA and 5%-fast for DP) and lead to lowest physiological responses at those combinations. Minimizing power fluctuations could be a main factor for preference and physiological response at 5%. However, these fluctuations are likely an outcome of more than one mechanism that is determining sub-technique preference. Preference for DP at 5% is quite likely because the contribution by the lower extremity is not impaired at relatively high speed, or at least to a far lesser extent than in DIA, by propulsion time and joint movement speed. Preference for DIA at 12% beyond DP is less clear. It is suggested that this is related to the reduced ability to utilize either the body's energy (provided by lower extremity) or active arm use in DP.

## Author contributions

CD, ØS, JD, GE designed the study; CD, JD, performed data collection; CD, GE performed data- and statistical- analysis; CD, ØS, JD, GE contributed to interpretation of the results; CD, GE wrote the draft manuscript; CD, ØS, JD, GE contributed to the final manuscript.

### Conflict of interest statement

The authors declare that the research was conducted in the absence of any commercial or financial relationships that could be construed as a potential conflict of interest.

## References

[B1] Ainegren M. Carlsson P. Tinnsten M. (2008). Roller ski rolling resistance and its effects on elite athletes' performance. Sports Eng. 11, 143–157. 10.1007/s12283-009-0016-5

[B2] Andersson E. Björklund G. Holmberg H. C. Ørtenblad N. (2016). Energy system contributions and determinants of performance in sprint cross-country skiing. Scand. J. Med. Sci. Sports. [Epub ahead of print]. 10.1111/sms.12666 26923666

[B3] Bilodeau B. Rundell K. W. Roy B. Boulay M. R. (1996). Kinematics of cross-country ski racing. Med. Sci. Sports Exerc. 28, 128–138. 10.1097/00005768-199601000-00024 8775365

[B4] Borg G. (1970). Perceived exertion as an indicator of somatic stress. Scand. J. Rehabil. Med. 2, 92–98. 5523831

[B5] Dahl C. (2016). A Biomechanical and Physiological Comparison of the Double Poling and Diagonal Stride Technique in Cross-Country Skiing. Master's thesis, University of Science and Technology, Norwegian.

[B6] Danielsen J. Sandbakk Ø. Holmberg H. C. Ettema G. (2015). Mechanical energy and propulsion in ergometer double poling by cross-country skiers. Med. Sci. Sports Exerc. 47, 2586–2594. 10.1249/MSS.0000000000000723 26110695

[B7] de Koning J. J. Foster C. Lampen J. Hettinga F. Bobbert M. F. (2005). Experimental evaluation of the power balance model of speed skating. J. Appl. Physiol. 98, 227–233. 10.1152/japplphysiol.01095.2003 15591304

[B8] de Leva P. (1996). Adjustments to Zatsiorsky-Seluyanov's segment inertia parameters. J. Biomech. 29, 1223–1230. 8872282 10.1016/0021-9290(95)00178-6

[B9] Ettema G. Lorås H. W. (2009). Efficiency in cycling: a review. Eur. J. Appl. Physiol. 106, 1–14. 10.1007/s00421-009-1008-7 19229554

[B10] Holmberg H. C. Lindinger S. Stöggl T. Eitzlmair E. Müller E. (2005). Biomechanical analysis of double poling in elite cross-country skiers. Med. Sci. Sports Exerc. 37, 807–818. 10.1249/01.MSS.0000162615.47763.C8 15870635

[B11] Hreljac A. (1995). Determinants of the gait transition speed during human locomotion: kinematic factors. J. Biomech. 28, 669–677. 10.1016/0021-9290(94)00120-S 7601866

[B12] Kehler A. L. Hajkova E. Holmberg H. C. Kram R. (2014). Forces and mechanical energy fluctuations during diagonal stride roller skiing; running on wheels? J. Exp. Biol. 217(Pt 21), 3779–3785. 10.1242/jeb.107714 25189366

[B13] Kvamme B. Jakobsen B. Hetland S. Smith G. (2005). Ski skating technique and physiological responses across slopes and speeds. Eur. J. Appl. Physiol. 95, 205–212. 10.1007/s00421-005-1332-5 16003540

[B14] Lindinger S. J. Göpfert C. Stöggl T. Müller E. Holmberg H. C. (2009). Biomechanical pole and leg characteristics during uphill diagonal roller skiing. Sports Biomech. 8, 318–333. 10.1080/14763140903414417 20169761

[B15] Millet G. Y. Hoffman M. D. Candau R. B. Clifford P. S. (1998). Poling forces during roller skiing: effects of grade. Med. Sci. Sports Exerc. 30, 1637–1644. 10.1097/00005768-199811000-00013 9813878

[B16] Pellegrini B. Zoppirolli C. Bortolan L. Holmberg H. C. Zamparo P. Schena F. (2013). Biomechanical and energetic determinants of technique selection in classical cross-country skiing. Hum. Mov. Sci. 32, 1415–1429. 10.1016/j.humov.2013.07.010 24071549

[B17] Péronnet F. Massicotte D. (1991). Table of nonprotein respiratory quotient: an update. Can. J. Sport Sci. 16, 23–29. 1645211

[B18] Sandbakk Ø. Ettema G. Holmberg H. C. (2012). The influence of incline and speed on work rate, gross efficiency and kinematics of roller ski skating. Eur. J. Appl. Physiol. 112, 2829–2838. 10.1007/s00421-011-2261-0 22127680

[B19] Sandbakk Ø. Holmberg H. C. Leirdal S. Ettema G. (2010). Metabolic rate and gross efficiency at high work rates in world class and national level sprint skiers. Eur. J. Appl. Physiol. 109, 473–481. 10.1007/s00421-010-1372-3 20151149

[B20] Stöggl T. L. Holmberg H. C. (2016). Double-poling biomechanics of elite cross-country skiers: flat versus uphill terrain. Med. Sci. Sports Exerc. 48, 1580–1589. 10.1249/MSS.0000000000000943 27031747

[B21] van Ingen Schenau G. J. Cavanagh P. R. (1990). Power equations in endurance sports. J. Biomech. 23, 865–881. 10.1016/0021-9290(90)90352-4 2211732

